# Neuromechanical force-based control of a powered prosthetic foot

**DOI:** 10.1017/wtc.2020.6

**Published:** 2020-10-23

**Authors:** Amirreza Naseri, Martin Grimmer, André Seyfarth, Maziar Ahmad Sharbafi

**Affiliations:** 1 Department of Mechanical Engineering, Tarbiat Modares University, Jalal al-Ahmad, Nasr, Tehran, Iran; 2 Lauflabor Locomotion Lab, Institute of Sport Science, Centre for Cognitive Science, Technische Universitat Darmstadt, Darmstadt, Germany

**Keywords:** force-based control, neuromechanical template model, neuromuscular model, prosthetic foot

## Abstract

This article presents a novel neuromechanical force-based control strategy called FMCA (force modulated compliant ankle), to control a powered prosthetic foot. FMCA modulates the torque, based on sensory feedback, similar to neuromuscular control approaches. Instead of using a muscle reflex-based approach, FMCA directly exploits the vertical ground reaction force as sensory feedback to modulate the ankle joint impedance. For evaluation, we first demonstrated how FMCA can predict human-like ankle torque for different walking speeds. Second, we implemented the FMCA in a neuromuscular transtibial amputee walking simulation model to validate if the approach can be used to achieve stable walking and to compare the performance to a neuromuscular reflex-based controller that is already used in a powered ankle. Compared to the neuromuscular model-based approach, the FMCA is a simple solution with a sufficient push-off that can provide stable walking. Third, to assess the ability of the FMCA to generate human-like ankle biomechanics during walking at the preferred speed, we implemented this strategy in a powered prosthetic foot and performed experiments with a non-amputee subject. The results confirm that, for this subject, FMCA can be used to mimic the non-amputee reference ankle torque and the reference ankle angle. The findings of this study support the applicability and advantages of a new bioinspired control approach for assisting amputees. Future experiments should investigate the applicability to other walking speeds and the applicability to the target population.

## 1 Introduction

During the last decades, advancements in modern medical lower-limb technologies have led to the emergence of assistive devices, such as exoskeletons, rehabilitation robots, and prostheses (Pillai et al., 2011). The significant key aspect of these devices is to restore users’ natural mobility. Some of the most important criteria used to examine the quality of wearable lower limb assistive devices are gait symmetry (Zanotto et al., 2014), the user’s energy expenditures (Au et al., 2009), and the adaptability to gait conditions (Gardiner et al., 2017).

It is evident that the metabolic cost of locomotion is higher in lower limb amputees, compared to unimpaired individuals (up to 60%), which can influence their mobility (e.g., walking speed) and quality of life (Colborne et al., 1992; Gailey et al., 1994; Schmalz et al., 2002).

Due to an insufficient energy return and a minimally effective push-off in passive prostheses, researchers developed active (or powered) prostheses. Moreover, while so far evidence is limited, they should restore the natural push-off power to improve the walking efficiency (Au et al., 2009; Ferris et al., 2012; Caputo and Collins, 2014; Quesada et al., 2016). Next, they should improve gait symmetry (Au et al., 2005; Ferris et al., 2012; Grimmer et al., 2017). In addition, the control of active prosthetic feet allows adaptation to different gaits, such as walking, running, ascending/descending stairs, and walking backward (Holgate et al., 2008; Grimmer et al., 2016).

Several control schemes have been proposed to mimic the biomechanics of an intact ankle joint by a prosthetic foot, which could also supplement passive compliance. Impedance control (Sup et al., 2009a), Quasi-stiffness control (Lenzi et al., 2014a), minimum jerk swing control (Lenzi et al., 2014b), and neuromuscular reflex control (Eilenberg et al., 2010) are compelling examples of these control techniques (see a review on different methods in Sharbafi et al. (2020)). The structure of these control schemes will be elaborated in the following.

Models with simple adjustable mechanical elements, such as springs (Grimmer, 2015) and dampers (Eslamy et al., 2013), can mimic the essential features of locomotion. Sup et al. (2008) showed that human joint torques can be sufficiently characterized by a simple impedance model comprised of a spring and a damper. However, Rouse et al. (2014) demonstrated that the impedance found at the ankle joint is changing even during the flat foot region of the stance phase. This shows that for ideal phase-based impedance control, the impedance needs to be tuned during the gait cycle (Rouse et al., 2014; Sup et al., 2008; Sup et al., 2009b). To control continuous gait and smooth transition between different speeds or environments, sensory input from lower limb kinematics (Holgate et al., 2009; Schmidt et al., 2017; Grimmer et al., 2019b) or kinetics (Eilenberg et al., 2010) is required.

It is still an open question of how to determine the desired impedance in locomotion as a dynamic procedure and how to adapt it based on human-robot-environment interaction. As an alternative, the neuromuscular models (Geyer and Herr, 2010), which are inspired by the human motor control, are used for control of prostheses (Eilenberg et al., 2010). Within neuromuscular models, intrinsic specifications of the actuators (e.g., stiffness and damping coefficients) are adjusted using length, velocity, and force feedback signals. A prosthetic foot that uses a neuromuscular model for control was developed at MIT (Eilenberg et al., 2010) and extended by Thatte and Geyer (2016).

The idea of utilizing such a neuromuscular model demonstrated the usefulness of understanding motor control in human locomotion to provide the required torque in assistive devices. Compared to the black box (e.g., trajectory-based) methods (Grimmer et al., 2017), white box (model-based) approaches could help to understand the underlying control principles of human locomotion. Therefore, the model-based approaches could potentially address the adaptability of the controller for different conditions. The main barrier for using such models to control assistive devices is their complexity and dependency on a large number of parameters. Thus, developing a simple and unique controller for lower limb exoskeletons (Quinlivan et al., 2017; Schmidt et al., 2017) or prostheses (Windrich et al., 2016) that can be used at different walking conditions is challenging.

To simplify neuromuscular models for control, neuromechanical template models were introduced (Sharbafi et al., 2020). The method is called *neuromechanical control* (Sharbafi et al., 2020), as mechanical elements (e.g., spring and damper) are used instead of muscle models. These mechanical elements are adjusted based on a simplified version of the neuromuscular model-based control (Prochazka et al., 2017) within the positive force feedback concept (Prochazka et al., 1997; Geyer et al., 2003). Force feedback is selected to tune the gait, as biomechanical studies on normal and pathological gaits demonstrated the importance of detecting body load (recorded by different types of receptors) to be used as proprioceptive feedback signals in locomotion control (Dietz et al., 1997; Duysens et al., 2000). The importance of force feedback was also demonstrated experimentally for compensatory leg muscle activation (Dietz et al., 1989). While in the neuromuscular control, muscle proprioceptive pathways are used to adjust the muscle activation (Geyer et al., 2003; Eilenberg et al., 2010), within the here presented FMCA (force modulated compliant ankle) control, the ground reaction force (GRF) is used to tune the mechanical impedance at the joint level. Therefore, the neuromechanical model replaces the proprioceptive signals (muscle force, length, and velocity) by the GRF and simplifies the neuromuscular model.

A similar neuromechanical control network was introduced by Sharbafi and Seyfarth (2015), where the GRF was used to tune the hip joint compliance in the so-called force modulated hip compliance model for controlling the human posture. This method was implemented on the LOPES II exoskeleton to assist human walking (Zhao et al., 2017). The results revealed that the force feedback system could reduce the human metabolic cost of walking (Zhao et al., 2019), as well as the consumed power in the assistive device. Inspired by these findings, the main contribution of the FMCA approach is to introduce a new technique to determine the desired ankle impedance using online GRF feedback. In agreement with this line of thought, previous work demonstrated that ankle torque is dependent on walking speed (Goldberg and Stanhope, 2013). Moreover, the evolution of the ankle torque is highly correlated to the corresponding speed-related GRF (Chiu et al., 2013; Nilsson and Thorstensson, 1989). This dependency supports the assertion of using GRF as a sensory signal to predict the ankle torque without obtaining the walking speed.

In this article, we will introduce the FMCA as a neuromechanical force-based control concept to control the ankle torque of a powered prosthetic foot. First, it was evaluated in theory if it is possible to mimic non-amputee reference ankle torques of different walking speeds with the FMCA control approach. Second, FMCA was implemented in a simulation model to evaluate the use in a prosthetic foot and to compare its functionality to a neuromuscular model (Geyer and Herr, 2010; Song and Geyer, 2013; Thatte and Geyer, 2016) and to non-amputee walking data (Lipfert, 2010). Finally, FMCA was tested on a powered prosthetic ankle during level-ground walking to evaluate the performance compared to non-amputee reference walking data.

## 2 Methods

### 2.1 FMCA model

**Figure 1.**
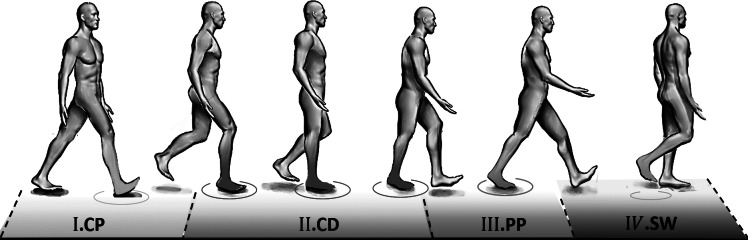
Phases during the gait cycle used to derive the ankle torque equation for level-ground walking.


In the FMCA, a unique function can be identified to approximate the required ankle torque at different walking speeds by employing the vertical GRF as the main sensory information. From reference unimpaired walking data of 21 subjects (Lipfert, 2010), we randomly selected 16 subject data as a training set to extract the FMCA parameters. The remaining five subjects’ data will be used as a test set for validation of the method. By defining a function of the ankle angle and the ankle angular velocity, which are modulated by the GRF, we introduce an adjustable impedance. With the help of these data, we were still not able to precisely estimate a human-like ankle torque, 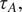 with a single equation. Therefore, to estimate the ankle torque with FMCA, the stance phase of walking was divided into three sub-phases: the controlled plantar flexion (CP), the controlled dorsiflexion (CD), and the powered plantar flexion (PP) (Figure 1). The separation of the stance period to sub-phases is a common approach for prosthesis control (Sup et al., 2009b; Rouse et al., 2014). The sum of the torque of these phases represents the ankle torque (Equation 1).

(1)

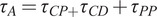



The CP is initiated when the heel touches the ground, and it lasts until the forefoot is on the ground (foot flat), which is in line with the first peak in ankle plantar flexion. The maximal weight acceptance, which is visible by the first vertical GRF peak, occurs at the end of this sub-phase. The CD starts at the end of the CP and continues until the heel-off, which initiates the PP. The ankle reaches the maximum dorsiflexion in the CD sub-phase. The second peak of the vertical GRF can be found at the end of this sub-phase. The PP starts with the heel-off and ends when the toe is leaving the ground. Therefore, the GRF can be used to detect different sub-phases of the stance phase.

The following procedure was used to estimate the ankle torque based on force modulated compliant ankle. We consider an individual equation for each of the sub-phases.

As in unassisted walking, where the GRF is the only force acting on the body, we estimated the ankle torque, 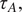 by Equation 2:
(2)

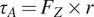

in which 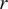 and 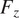 are the moment arm and vertical ground reaction force, respectively. As 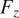 is pointed at the CoP (center of pressure) and 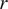 is the distance from the ankle joint to 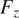 , changing the moment arm is equal to changing the CoP. In the FMCA approach, the desired ankle torque is given by a linear function of the GRF based on Equation 2. The idea is to estimate the moment arm as a polynomial function of the ankle angle 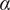 and the ankle angular velocity 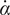 (Equation 3).
(3)

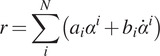



The coefficients ( 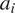 , 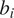 , and 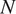 ) are identified in an iterative process using curve fitting and MATLAB optimization toolbox (Levenberg–Marquardt algorithm, nonlinear solver). The goal was to find the minimal model (lowest complexity of the equation) that can precisely approximate the moment arm at three different speeds of human walking. These speeds are categorized into slow (50% PTS, PTS is defined as the preferred transition speed between walking and running), moderate (75% PTS), and fast (100% PTS). Finally, the values of all three equations were interpolated to get a unique equation for the sufficiently precise approximation of the ankle torque at all walking speeds. This procedure is performed similarly for CP, CD, and PP.

Assume 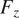 is fixed (e.g., in standing still) and set to the constant 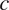 . Then, the ankle torque of Equation 3 can be described as ankle compliance (Equation 4).
(4)

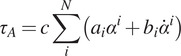



**Figure 2.**
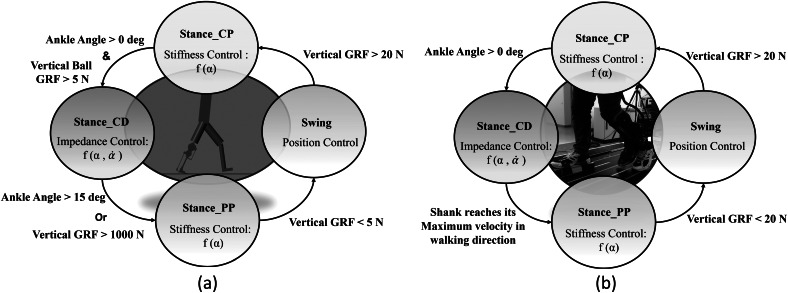
The finite-state machine of the force modulated compliant ankle control for controlling the prosthetic foot. The ankle angle and the ankle angular velocity are shown by α and 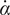 . (a) Implementation in the neuromuscular simulation model. (b) Implementation in the powered prosthetic foot. The shank velocity from the gyro is a filtered signal that shifts the negative peak from the previous stride to about 59% of the gait cycle of the following stride.

This means that Equation 3 can be interpreted as a force-modulated compliant ankle (FMCA) mechanism. In other words, the ankle actuator can be represented as a nonlinear compliant element (with springs and dampers), which is tuned by the GRF signal. The FMCA control is implemented by identifying the coefficients of Equation 3 separately for CP, CD, and PP. In addition, a finite-state machine (Figure 2) was used to define the guard conditions for the transition between the states within the gait cycle. This figure depicts the switching conditions between CP, CD, PP, and the swing phase. It is shown that a (non)linear spring is sufficient to model the adjustable compliance in the PP and the CP sub-phases (see Section 3.1 for details of the identified equations).

### 2.2 Simulation

Simulations were performed in the Simulink toolbox of MATLAB. The simulation presented here uses a modified neuromuscular model of Geyer and Herr (2010), including the prosthetic foot for one leg, developed by Thatte and Geyer (2016). To control the prosthetic foot, the neuromuscular model utilizes an ankle plantar flexor, which comprises a Hill-type muscle model with positive force reflex, to mimic the behavior of an intact ankle. For our investigation, the reflex-based prosthetic foot control (Thatte and Geyer, 2016) was modified to include the FMCA control. Furthermore, the reflex-based foot control was used as a reference for the validation of the FMCA control. When modifying the reflex-based model, everything but the control of the prosthetic foot ankle joint was kept the same as in the original study (Thatte and Geyer, 2016). As stated in Section 2.1, the gait cycle was divided into three states for the stance phase and one state for the swing phase. Figure 2a shows the state machine for the simulation model. As shown in this figure, the switching conditions are defined based on two signals: vertical ground reaction force and ankle angle. Simulations were performed for 20 seconds. During this time, both models predicted 14 strides with the simulated prosthetic limb. The last 11 strides were used for the comparison of the models. In Section 3.2, we demonstrate the performance of these two models to compare the FMCA and neuromuscular models’ capability in controlling the prosthetic foot. The resulting torque and angle of the prosthetic foot beside the GRF were considered for comparison.

### 2.3 Walking experiment

**Figure 3.**
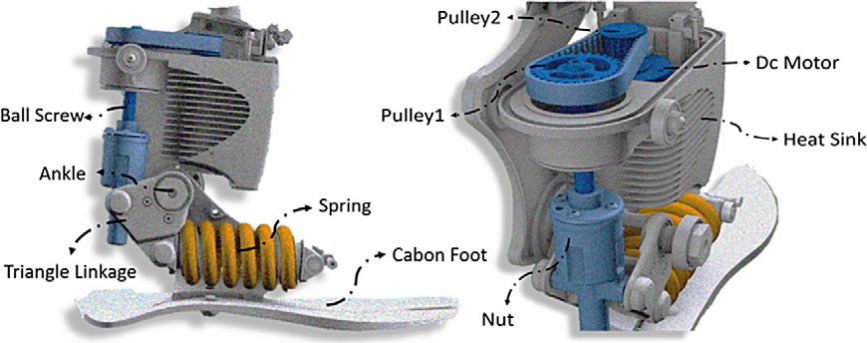
CAD representation of the Ruggedized Odyssey Ankle prosthetic foot (Ward et al., 2015; CDMRP, 2018).

After analyzing the FMCA control strategy with the neuromuscular simulation model, FMCA was implemented in the Ruggedized Odyssey Ankle (ROA, Figure 3) to perform a level walking experiment. The walking experiment was conducted with one non-amputee subject without any known gait-related impairments (age: 24 years, mass: 72 kg, height: 1.72 m). A powered prosthetic ankle was mounted on the left shank with the help of a bypass-adapter (Figure 2b). The contralateral leg length was increased by additional foam, mounted below the shoe, to match the leg length of the left leg during standing. The study was approved by the Institutional Review Board of the Technische Universität Darmstadt. The subject provided written informed consent before participation. For sensory feedback, vertical GRF was measured individually for both limbs by an instrumented treadmill (ADAL-WR, HEF Tecmachine, Andrezieux Boutheon, France). During the experiment, the subject walked at 75% PTS for 4 minutes. The generated torque and angle in the prosthesis and the GRF were measured for further analyses.

#### 2.3.1 Powered prosthetic foot

A tethered powered prosthetic foot (ROA, SpringActive, US) was used to evaluate FMCA (Figure 3). The prosthetic foot is an updated version of the Walk-Run ankle, previously used to investigate walking and running (Grimmer et al., 2016; Grimmer et al., 2017). Similarly, ROA uses a DC motor to rotate a ball screw, which moves a carbon foot via a linkage anchored at the ankle joint. A series steel spring is located between the ball screw and the carbon foot. ROA comprises a motor encoder to measure the motor position and an ankle encoder to measure the ankle angle. Furthermore, it includes an inertial measurement unit (IMU) placed inside the heat sink.

**Figure 4.**
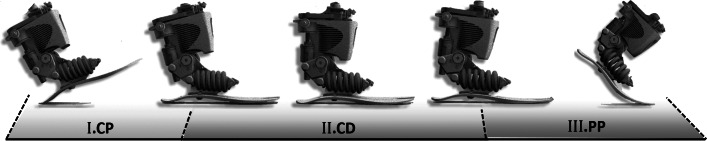
Working principle of the Ruggedized Odyssey Ankle prosthetic foot during the stance phase (Ward et al., 2015; CDMRP, 2018).

Figure. 4 shows the ROA activity during the stance phase of level-ground walking. During the stance, the ROA can modulate the ankle joint impedance. During the CP, ROA can store energy in the heel spring, and the motor of the powered foot can act as a damper to absorb the shock generated by the weight applied to the prosthetic limb. At the beginning of the CD, the prosthetic carbon foot exploits the stored energy to facilitate the shank’s rotation. During CD, the load due to the subject and the motor can be used to store energy in the steel spring and the forefoot carbon spring to support the following push-off. Furthermore, the motor can be used to modulate damping related effects. During PP, the steel spring and the carbon forefoot spring can release the stored energy (positive work), and the motor can provide positive work. The combined positive ankle work is used to move the center of mass and the swing leg forward.

When the foot leaves the ground (swing phase), we apply a position control by driving the motor to set the ankle angle to a fixed predefined configuration 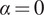 .

To mimic the ankle torque and the ankle angle of the ROA during walking, we propose the FMCA scheme. Similar to the simulation model, the stance phase was divided into three sub-phases. Then, a finite-state machine (depicted in Figure 2b) is designed to determine switching between these sub-phases and to the swing phase. The sagittal shank angular velocity, which is sensed by the gyro sensor of the IMU, replaced the GRF as a guard condition for the transition from CD to PP. Although the vertical GRF from the instrumented treadmill could have been used, we employed the angular velocity data for detecting the PP sub-phase to compare the results with the outcomes from trajectory-based methods used in previous works (Grimmer et al., 2016; Grimmer et al.,^2017^). This comparison is not presented in this work. FMCA was used to define the ankle torque within the whole stance phase. This includes different GRF-based torque formulations in the three sub-phases of the stance. The energy injection is mainly performed in the PP sub-phase, which is the most important period for assisting gait with the powered foot.

### 2.4 Data evaluation

For all evaluations, the data from FMCA are compared to human non-amputee reference walking data. The reference data contain the mean and standard deviation of walking biomechanics (ankle torque, ankle angle, and vertical GRF) from 21 subjects (Lipfert, 2010) walking at 50% PTS, 75% PTS, and 100% PTS. For all comparisons, the ankle torque and the vertical GRF were normalized to the body weight. Furthermore, the ankle torque was normalized to the leg length.

#### 2.4.1 FMCA model

After tuning and optimizing the equations for FMCA to match the reference ankle torque data, a comparison to the reference data was performed for two data set. (a) A set of data (16 sample data randomly among 21 subjects) was used to optimize the FMCA parameters, and (b) a set of data (five remaining sample data) was used to evaluate the FMCA approach in predicting ankle torque. A second-order Butterworth filter with a cut-off frequency of 7 Hz was utilized to smoothen the FMCA torque during the whole stance. For comparison, Pearson’s correlation value and the mean absolute difference of individual FMCA-based torque (just stance) predictions were compared with the mean of the reference data. Correlation values have a range from 0% to 100%, with 100% indicating a perfect linear correlation. With FMCA, we aim for a correlation of close to 100% for the tested three walking speeds. The mean absolute difference was normalized to the peak of the average torque from the reference data for each speed.

#### 2.4.2 Simulation

For comparison, the mean of the ankle angle, the ankle torque, and the vertical GRF were determined based on 11 strides. For FMCA and the reflex model, the ankle angle was determined by a revolute joint encoder. Both methods used force sensors at the heel and the ball to determine the GRF. The ankle torque was determined by the ankle torque equations (Equations 1, 5–7) based on FMCA and the artificial muscle reflexes of the neuromuscular model (Thatte and Geyer, 2016). Walking with FMCA is compared to the reference data (Lipfert, 2010) and the reflex-based model (Thatte and Geyer, 2016). Pearson’s correlation value was determined to evaluate for a linear relationship between the torque based on FMCA and the reference data and based on the reflex-based model and the reference data. We target a similar or increased Pearson’s correlation with the FMCA control, compared to the reflex-based control.

#### 2.4.3 Walking experiment

**Figure 5.**
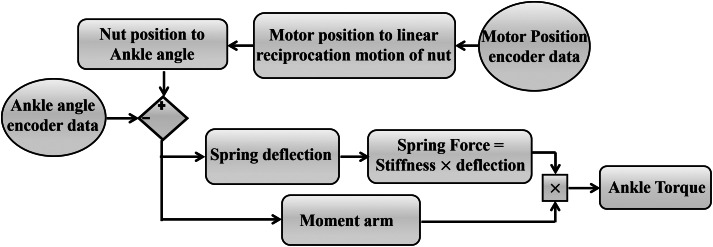
The flowchart of geometry-based ankle torque calculation.

A level treadmill walking experiment was performed to extract the walking biomechanics with the ROA, controlled by the FMCA method. For the comparison to the non-amputee reference data (Lipfert, 2010), the mean of the ankle angle, the ankle torque, and the vertical GRF were calculated based on 13 strides. These strides are selected from the steady-state walking. The ankle angle of the ROA was determined by the ankle encoder. The series elastic actuator of the ROA has two degrees of freedom in the stance phase; hence the ankle torque of the ROA was determined based on the ankle angle and the motor position (Figure 5). To compute the actual ankle torque of the ROA, we considered the position source role of the motor. Utilizing the pulley ratio in addition to the screw pitch, the geometry-based angle change can be determined. Comparing this angle to the actual ankle angle measured by the ankle encoder results in both the spring deflection and the moment arm deviation with respect to their neutral length. Therefore, the actual ankle torque could be calculated during the stance phase. The vertical GRF was determined by the sensors in the instrumented treadmill for each leg, separately. Pearson’s correlation value and the mean absolute difference between the torque based on FMCA and the reference data were determined.

## 3 Results

### 3.1 FMCA model

A single equation for each of the sub-phases of stance was determined, as explained in Section 2.1. Furthermore, we had to define the zero angle of the ankle at the perpendicular position of the shank and the foot.

For the first sub-phase (CP), we found that the moment arm only relies on the ankle angle. Thus, a first-order equation (Equation 5) could be considered to match the desired ankle torque.
(5)

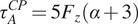



For the second sub-phase (CD), we found that not only the anterior/posterior movement of 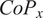 depends on the ankle angle but also the ankle angular velocity should be considered. Thus, a second-order equation of the angular velocity was added to the linear equation of the angle to mimic the ankle torque (Equation 6).
(6)

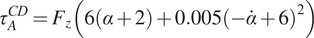



For the PP sub-phase, we discovered that the moment arm is only a function of the ankle angle. For preventing any sudden dropping effect in the ankle torque, when the ankle torque reaches its maximum at the end of CD, a cubic function of the ankle angle was used to estimate the ankle torque (Equation 7).
(7)

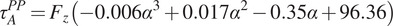



**Figure 6.**
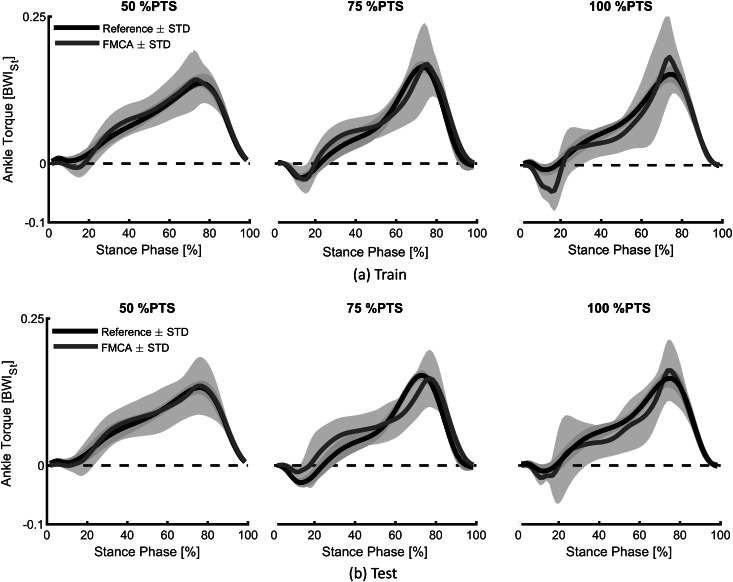
The ability of the force modulated compliant ankle approach in predicting human ankle torque during the stance phase of walking at three different speeds (preferred transition speed). The data set of 21 subjects, adopted from Lipfert (2010) is divided into (a) a training set with 16 randomly selected subjects and (b) a test set with the remaining five subjects. The solid line and shaded region show the mean and the standard deviation of multiple strides and multiple subjects, respectively.

**Figure 7.**
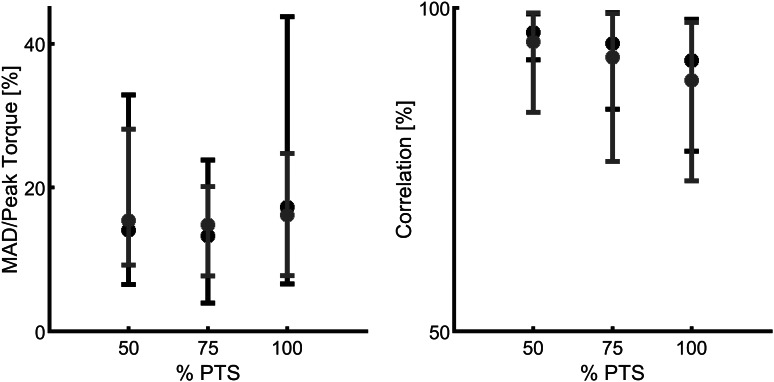
Quantitative representation of the ankle torque approximation with the force modulated compliant ankle method, using the mean absolute difference (MAD) and the correlation values. The data set includes 21 subjects walking at three different speeds (preferred transition speed), adopted from Lipfert (2010). Black and red colors show the results of training (16 subjects) and test (5 subjects), respectively. The MAD data are normalized to the peak of the average torque from the reference data.

The extracted unique FMCA model, using the training set with 16 subjects’ data (Lipfert, 2010), was utilized to predict the human ankle torque at three different speeds. First on the same data set (Figure 6a) and then on the test data set with five subjects (Figure 6b). Comparison of reference data and FMCA prediction in Figure 6a shows appropriate matching in all three speeds, while the approximation quality slightly decreases by increasing walking speed. To provide a more quantitative evaluation measurement, we calculated the correlation and the mean absolute difference (MAD) between the FMCA and the reference values for each subject from the training set. Figure. 7 reveals that the average correlation (of 16 subjects) is more than 91%, and the average MAD is less than 18% of the reference peak torque for all speeds. Correlation above 94% and normalized MAD less than 15% for the low and medium walking speeds confirm perfect estimation in these speeds, as identified in Figure 7.

To verify the predictive power of the proposed method, we applied the same FMCA formulation (with the same coefficients) to approximate reference ankle torque from the test data set. Figure. 6b demonstrates that the quality of predicting the test data is quite comparable with that of the training set (Figure 6a). More quantitatively speaking, the average correlation is more than 87%, and the average MAD is less than 17% of the peak torque for all speeds, as shown in Figure 7. As can be seen in this figure, the prediction quality is slightly less for the test data except for the MAD at 100% PTS, which is only 1% lower than this value for training data. In general, it is fair to say that the comparison between the training and test data sets confirm cross-validation of the predictive ability of the FMCA.

### 3.2 Simulation

In order to evaluate the control quality of the FMCA, we considered the body CoM (center of mass) and GRF as two measures for kinematic and kinetic behavior of the whole body (macroscopic level). Then, the ankle torque and angle were analyzed and compared to the reference data. In all cases, we compared the reflex control and the FMCA with respect to the similarity of the resulting behavior to the reference healthy walking data from Lipfert (2010).

**Figure 8.**
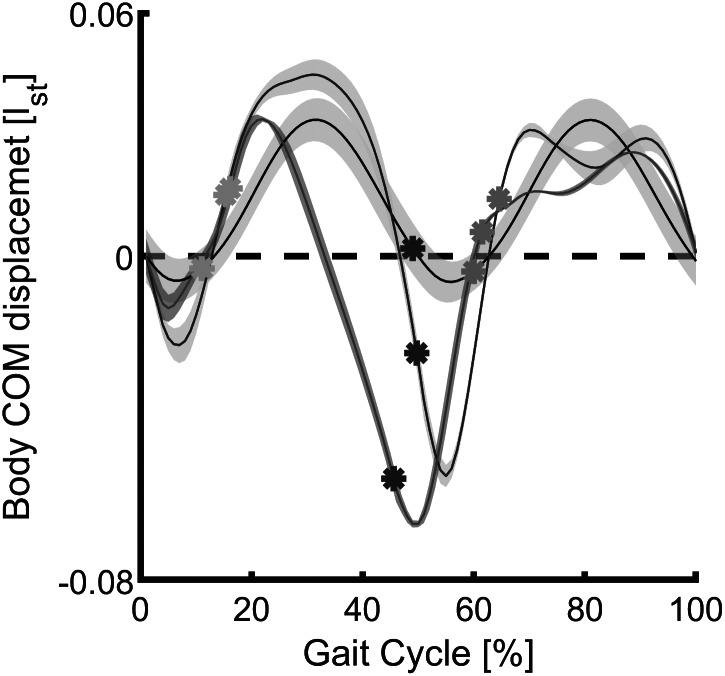
Mean and standard deviation of the body center of mass displacement in the sagittal plane during 11 strides (75% preferred transition speed) for the force modulated compliant ankle based control (red) and the reflex-based control (blue). The unimpaired reference data (black) include the mean and the standard deviation of multiple strides and multiple subjects (Lipfert, 2010). Within the simulation, the data are shown for one gait cycle (starting at the heel strike) of the prosthetic limb. Yellow, purple, and green circles represent the contralateral limb toe-off, contralateral limb heel strike, and the ipsilateral limb toe-off, respectively.

The general trend of the vertical CoM displacement (Figure 8) of both simulations (using reflex-based and FMCA) is similar to the reference with one negative and one positive peak at each step. One discrepancy is the inequivalent CoM height at the touchdown of different legs (0% of the gait cycle and purple dots). This is worse in the FMCA controlled case. In the first single support phase (0% to yellow circle), the FMCA control is more similar to reference with lower downward excursion than reflex control. The following double support (from yellow to purple circle) in all cases is concave downward. In the next step, the lowered CoM in amputee walking simulations should be compensated by the intact limb. The double hump pattern in the second single support achieved by reflex control is different from the reference concave downward pattern, while the FMCA also has a single peak.

**Figure 9.**
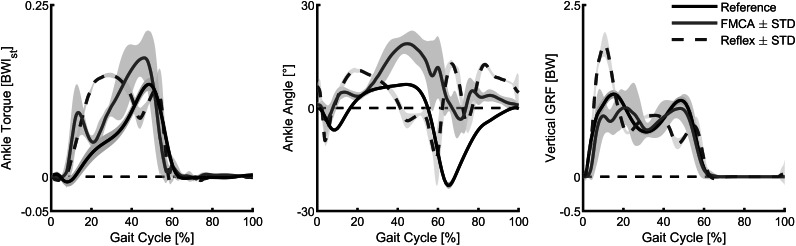
Ankle torque, ankle angle, and vertical ground reaction force of walking (75% preferred transition speed) for a reflex-based control (blue, simulation), the force modulated compliant ankle based control (red, simulation), and unimpaired reference data (black, experiment). The simulation data include the mean and standard deviation of 11 strides. The reference data include the mean and the standard deviation of multiple strides and multiple subjects (Lipfert, 2010). Positive torque is an extension torque. Positive angles represent dorsiflexion. The zero-ankle angle is the perpendicular position of the shank and the foot.

When comparing the ankle torque of FMCA and the reflex-based control to those of the reference data (Lipfert, 2010), we found increased torque values within the stance phase for both of them (Figure 9, left). The peak torque during the PP was highest for FMCA. In comparison, with the reflex-based control, next to the peak during the PP, a second peak at about 30% of the gait cycle was observed. Compared to the reference data, the early peak of the reflex-based control is higher, and the late peak of the reflex-based control is smaller. The correlations were determined to evaluate the similarity of the ankle torque of both control approaches (FMCA and reflex-based) and the reference data. Using the Pearson method, we found a correlation of 90% for FMCA and a correlation of 76% for the reflex-based control.

Compared to the reference walking data and the FMCA-based control, the angle of reflex-based control starts plantar flexion early (Figure 9, middle). On the other hand, FMCA has larger dorsiflexion compared to the reference and the reflex-based control, while the timing of this peak in FMCA matches better to the reference data than the reflex-based control. During the swing, both FMCA and reflex-based have increased dorsiflexion compared to the reference data, while the pattern and the timing of the FMCA control match the reference data better than the reflex-based control. Since the neuromuscular model composition (Thatte and Geyer, 2016) does not exactly represent the human body composition (Lipfert, 2010), we do not expect completely similar angle patterns. However, the correlation between the outcomes of FMCA and the experimental data is 56%, while this value for the neuromuscular control is 5%.

In both simulation models, the vertical GRF has three peaks, compared to the two peaks of the walking reference data (Figure 9, right). The first peak of the reflex model is much larger, and the following two peaks have a different timing and lower amplitude compared to the reference data. Moreover, while the peaks are lower for FMCA, they match the reference data better, especially during push-off, compared to the reflex model. The correlations between the GRF from the reference data and FMCA and the reference data and the reflex-based control are 95% and 85%, respectively.

In summary, when simulating walking with the FMCA control, the ankle torque, the ankle angle, and the vertical GRF (Figure 9) are more similar to the non-amputee reference data compared to the reflex-based control.

### 3.3 Walking experiment

**Figure 10.**
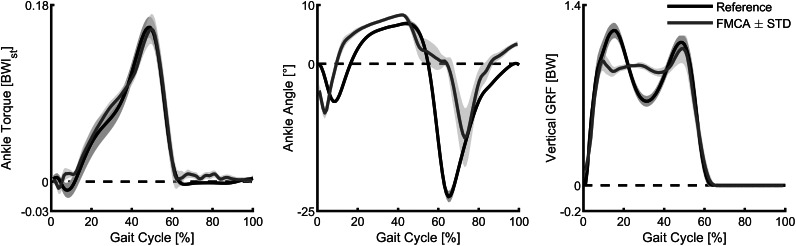
Ankle torque, ankle angle, and vertical ground reaction force of walking (75% preferred transition speed) for the subject wearing the powered prosthetic foot controlled with force modulated hip compliance approach (red) and unimpaired reference data (black). The prosthetic foot data include the mean and the standard deviation of 13 strides of the prosthetic side. The reference data include the mean and the standard deviation of multiple strides and multiple subjects (Lipfert, 2010). Positive torque is an extension torque. Positive angles represent dorsiflexion. The zero-ankle angle is the perpendicular position of the shank and the foot.

Similar to the simulations, we compared the ankle torque, the ankle angle, and the vertical GRF of the assisted walking with FMCA to the walking reference data (Figure 10). The ankle torque with FMCA has a similar shape and magnitude as the reference walking data (Figure 10, left). The correlation between the ankle torque with FMCA and the reference ankle torque is 99%.

The ankle angle with FMCA matches the reference data during most of the stance phase. During the late stance and the early swing, the plantar flexion is delayed (Figure 10, middle). As a result, the correlation between them is 70%.

The vertical GRF with FMCA shows a reduction in the first GRF peak and an increased GRF during midstance, compared to the reference walking data (Figure 10, right). The second GRF peak almost matches the reference data. Moreover, while showing differences, a correlation of 96% was achieved between both GRF patterns.

The mean absolute difference (between FMCA and reference) for the three variables are: normalized ankle torque = 0.0052, ankle angle = 4.5°, normalized vertical GRF = 0.076. These numbers are less than 4%, 20%, and 7% of the peak values of torque, angle, and GRF, respectively.

## 4 Discussion

In this paper, a new control method that uses an abstract (template model-based) description of the biological neuromuscular system, the neuromechanical FMCA model, was introduced. The FMCA is used to approximate the human ankle torque in walking. Our study includes two steps: (a) the identification of the FMCA parameters, based on healthy human walking data, and (b) the validation of the FMCA approach. This second step comprises: (a) cross-validation using a set of unseen data from experiments similar to step 1, (b) a simulation study with comparison to a neuromuscular controller, and (c) prosthesis walking experiments involving one individual subject to confirm the functionality of the method. In the following, we reflect on the approach of the FMCA control and discuss the achievements and shortcomings of this approach.

### 4.1 FMCA model

The FMCA control approach can also be described as variable impedance control. Here, a combination of linear and nonlinear springs and dampers represents the desired variable impedance at the different sub-phases of the stance phase. In (Rouse et al., 2014), the ankle impedance was analyzed during the CD (flat foot) sub-phase, and it was shown that the impedance was adapted even in such a short period with unchanged dynamics. In other studies, changing impedance by switching between gait phases (e.g., three sub-phases in stance phase) was utilized to generate human-like motor control in prostheses (Sup et al., 2008; Sup et al., 2009a). Therefore, the desired impedance depends not only on the gait conditions (e.g., speed) but also to a variable function of the gait phase during each stride. In addition, the difference between individual gait patterns will complicate developing a phase-based impedance control. Our GRF-based impedance calculation provides the possibility to adapt the ankle torque online at each time-instance to different gait conditions and also for individualization. Thus, the impedance is not predefined, which could be of advantage for control.

The goal of the FMCA is not minimizing the overall error between the reference and predicted ankle torques. In that respect, it is possible to find a lower-order polynomial function of the ankle angle to predict the torque pattern during the PP sub-phase (push-off). However, the proposed nonlinear spring (Equation 7) could provide a perfect energy injection during push-off at different walking speeds (Figure 6). This is an appropriate initial guess, and the coefficients could be fine-tuned individually for different subjects (not analyzed in this paper).

With the help of the identified FMCA model parameters (the vertical GRF, the ankle angle, and the ankle angular velocity), the FMCA approach was able to estimate the required ankle torque throughout the gait cycle. We found that, in theory, FMCA can be used to match the human reference ankle torque of walking at different speeds with a mean absolute difference of less than 17% of the peak torque. This finding was highly promising regarding the usability of the approach. Based on the predictive ability of the proposed approach, which is confirmed by the cross-validation using training and test data sets, we expect that the FMCA method can also be easily adapted for different gait conditions (e.g., walking speeds) with a minimal parameter adjustment. The adaptability of the ankle behavior, based on the vertical GRF (within FMCA), confirms that the GRF has an essential role in controlling legged locomotion. In continuation, the control approach was validated by the simulation and the experiment.

### 4.2 Simulation

The purpose of performing the simulation was to evaluate the neuromechanical template control regarding the applicability for generating stable walking. Furthermore, we wanted to compare the performance with an existing neuromuscular model (reflex-based, (Thatte and Geyer, 2016)), which is the most accepted model to predict the general behavior (motor control) of human walking. The neuromuscular model demonstrated stable human-like walking for a range of walking conditions (Eilenberg et al., 2010) and different lower limb joints (Thatte and Geyer, 2016). We used the neuromuscular model and replaced the control of the ankle with the neuromechanical FMCA control approach. Following, the neuromuscular model and non-amputee walking data were used as a reference to evaluate the FMCA control approach.

We found that similar to the reflex-based model; the FMCA model can generate stable walking at 1.3 m/s (75% PTS). For both models, differences in the ankle torque, the ankle angle, and the vertical GRF exist, compared to the non-amputee reference data. Interestingly, differences between the reflex-based and the FMCA models were also identified for the swing phase, while the swing phase control was the same for both models. Thus, the stance seems to have a major impact on swing phase dynamics. Based on the data, the huge peak torque during the late stance with FMCA seems to result in a closer match to the second vertical GRF peak of the non-amputee reference data. In comparison, a lack of human-like push-off torque seems to cause a reduced second vertical GRF peak for the reflex-based model. Moreover, while it is hard to compare, the FMCA model is a much simpler control solution, and it demonstrated improved push-off performance compared to the reflex-based control. However, the reflex-based control already demonstrated to be able to provide similar amounts of ankle work like non-amputees, during walking at different slopes (Eilenberg et al., 2010). Generating stable amputee walking (as shown in the attached video) with a comparable kinematic and kinetic behavior to experimental data validates the FMCA method (as the second step of the study).

### 4.3 Walking experiment

When comparing walking with the FMCA controlled powered prosthetic ankle to the reference non-amputee walking data, we found differences mainly for the ankle angle and the vertical GRF. We believe that part of the difference is due to the different leg configuration (e.g., elevated non-prosthetic foot, bypass at prosthetic foot). Independent of the leg configuration, we found that the ankle angle during the late stance had a delayed plantar flexion. As the plantar flexion is larger after the toe-off, we believe that the torque created by the FMCA control during the PP was not sufficient to allow a continuous increase in the plantar flexion. As the desired torque values were achieved, we believe that individual adaptations of the ankle torque, based on the ankle angle, are required. Still, the similarity between the measured ankle torque from the assisted walking experiment and reference data is remarkable (99% correlation). The measured ankle angle and GRF profiles of the prosthesis also qualitatively match those of the reference walking, as shown in Figure 10. This is supported quantitatively by the calculated correlation (70% for angle and 96% GRF) and the mean absolute difference for each of these shown graphs.

## 5 Conclusions

This study found that, in theory, it is possible to mimic the ankle joint torque of different walking speeds, regardless of changing the control parameters, based on the vertical ground reaction force with the FMCA concept. We verified the performance of the proposed model first, with cross-validation using human unimpaired walking data, then using a walking simulation model, finally, in a pilot prosthesis walking experiment. It was shown that the FMCA method could emulate the reference ankle torque with acceptable precision. When comparing the outcomes of the simulation and walking experiment to the reference non-amputee level walking data, differences in the ankle torque, the ankle angle, and the vertical ground reaction force were much smaller for the experiment compared to the simulation. In the simulation, the correlations to the reference were reduced for the FMCA control (neuromechanical), compared to the reflex-based control (neuromuscular). The results support that the neuromechanical FMCA template model, which is a simplified abstraction of a complex neuromuscular model, can provide a control solution to mimic the dynamic behavior of the human ankle joint during level walking. In the control concept and implementation level, the main differences of the FMCA to the neuromuscular control are (a) using GRF to tune joint muscle-like properties (impedance) instead of the muscle reflex (force, length, or velocity) and (b) simplifying the muscle by an adjustable impedance. Therefore, FMCA introduces a simpler formulation with new sensory information (GRF) as the reflex signal. Indeed, both methods try to predict human motor control for generating a model-based prosthesis control.

Therefore, giving the FMCA-generated torque patterns to a powered prosthetic foot provides the possibility to get independent of the time information, which is required in the trajectory-based methods. Furthermore, the force feedback includes information about the locomotion status, which is absent in methods using time-based trajectories. For example, perturbations during walking are reflected in the GRF, which, in return, can be used to modulate the torque output of the ankle to support a proper reaction. In this regard, the idea of developing a controller (with fixed parameters), which can adapt to different gait conditions (and perturbations), can be considered as an outlook of the FMCA design concept. The adaptability of FMCA is partially supported by predicting the human ankle torque as a function of the GRF and the ankle angle for five different subjects at three different speeds.

The presented experimental results are from an early stage of this research. Improvements in the FMCA control might be required for a closer match to the reference non-amputee ankle angle during late stance. So far, vertical GRF from the treadmill was used to realize the control approach. For an autonomous system, force sensors in the shank, as exploited in the C-Leg (Carroll and Edelstein, 2006) or in (Schuy et al., 2014), could be used to estimate the GRF. The following experiments should include more subjects and walking speeds to get a better idea if it is required to individualize FMCA-related parameters to each subject and to investigate the performance at different speeds. Finally, the ability of the FMCA to improve amputee gait or the gait of other mobility-impaired populations with exoskeletons (Grimmer et al., 2019a) in parameters such as preferred walking speed, gait symmetry, or metabolic cost has to be evaluated.

## Supplementary material

For supplementary material accompanying this paper visit https://doi.org/10.1017/wtc.2020.6. click here to view supplementary material

## References

[r1] Au SK, Bonato P and Herr H 2005 An EMG-Position Controlled System for An Active Ankle-Foot Prosthesis: An Initial Experimental Study, 9th International Conference on Rehabilitation Robotics, 2005. ICORR 2005. Chicago: IEEE, pp. 375–379. Accessed date: July 1, 2005. Available at https://ieeexplore.ieee.org/document/1501123.

[r2] Au SK, Weber J and Herr H 2009 Powered ankle--foot prosthesis improves walking metabolic economy. IEEE Transactions on Robotics 25(1), 51–66.

[r3] Caputo JM and Collins SH 2014 Prosthetic ankle push-off work reduces metabolic rate but not collision work in non-amputee walking. Scientific Reports 4, 7213. 25467389 10.1038/srep07213 PMC4252906

[r4] Carroll K and Edelstein JE 2006 Prosthetics and Patient Management: A Comprehensive Clinical Approach. Thorofare: Slack Incorporated.

[r5] CDMRP 2018 *SpringActive Odyssey Ruggedized Ankle Prosthesis*. [Video] YouTube: https://youtu.be/4WwOj4sj6QM. Congressionally Directed Medical Research Programs (uploaded 25 April 2018).

[r6] Chiu M-C, Wu H-C and Chang L-Y 2013 Gait speed and gender effects on center of pressure progression during normal walking. Gait & Posture 37(1), 43–48. 22824680 10.1016/j.gaitpost.2012.05.030

[r7] Colborne GR, Naumann S, Longmuir PE and Berbrayer D 1992 Analysis of mechanical and metabolic factors in the gait of congenital below knee amputees. A comparison of the SACH and Seattle feet. American Journal of Physical Medicine & Rehabilitation 71(5), 272–278. 1388973 10.1097/00002060-199210000-00004

[r8] Dietz V, Horstmann G, Trippel M and Gollhofer A 1989 Human postural reflexes and gravity—an under water simulation. Neuroscience Letters 106(3), 350–355. 2601889 10.1016/0304-3940(89)90189-4

[r9] Dietz V, Leenders K and Colombo G 1997 Leg muscle activation during gait in Parkinson’s disease: influence of body unloading. Electroencephalography and Clinical Neurophysiology/Electromyography and Motor Control 105(5), 400–405. 9363006 10.1016/s0924-980x(97)00042-8

[r10] Duysens J, Clarac F and Cruse H 2000 Load-regulating mechanisms in gait and posture: comparative aspects. Physiological Reviews 80(1), 83–133. 10617766 10.1152/physrev.2000.80.1.83

[r11] Eilenberg MF, Geyer H and Herr H 2010 Control of a powered ankle–foot prosthesis based on a neuromuscular model. IEEE Transactions on Neural Systems and Rehabilitation Engineering 18(2), 164–173. 20071268 10.1109/TNSRE.2009.2039620

[r12] Eslamy M, Grimmer M, Rinderknecht S and Seyfarth A 2013 Does It Pay to Have A Damper in a Powered Ankle Prosthesis? A Power-Energy Perspective, 2013 IEEE 13th International Conference on Rehabilitation Robotics (ICORR). Seattle: IEEE, pp. 1–8. Available at https://ieeexplore.ieee.org/document/6650362. 10.1109/ICORR.2013.6650362 24187181

[r13] Ferris AE, Aldridge JM, Rábago CA and Wilken JM 2012 Evaluation of a powered ankle-foot prosthetic system during walking. Archives of Physical Medicine and Rehabilitation 93(11), 1911–1918. 22732369 10.1016/j.apmr.2012.06.009

[r14] Gailey RS, Wenger MA, Raya M, Kirk N, Erbs K, Spyropoulos P and Nash MS 1994 Energy expenditure of trans-tibial amputees during ambulation at self-selected pace. Prosthetics and Orthotics International 18(2), 84–91. 7991365 10.3109/03093649409164389

[r15] Gardiner J, Bari AZ, Howard D and Kenney L 2017 Transtibial amputee gait efficiency: energy storage and return versus solid ankle cushioned heel prosthetic feet. Journal of Rehabilitation Research and Development 53(6), 1133–1138. 10.1682/JRRD.2015.04.0066 28355033

[r16] Geyer H and Herr H 2010 A muscle-reflex model that encodes principles of legged mechanics produces human walking dynamics and muscle activities. IEEE Transactions on Neural Systems and Rehabilitation Engineering 18(3), 263–273. 20378480 10.1109/TNSRE.2010.2047592

[r17] Geyer H, Seyfarth A and Blickhan R 2003 Positive force feedback in bouncing gaits? Proceedings of the Royal Society of London. Series B: Biological Sciences 270(1529), 2173–2183. 10.1098/rspb.2003.2454 PMC1691493 14561282

[r18] Goldberg SR and Stanhope SJ 2013 Sensitivity of joint moments to changes in walking speed and body-weight-support are interdependent and vary across joints. Journal of Biomechanics 46(6), 1176–1183. 23374276 10.1016/j.jbiomech.2013.01.001 PMC3605195

[r19] Grimmer M 2015 Powered Lower Limb Prostheses. Darmstadt: Technische Universität.

[r20] Grimmer M, Holgate M, Holgate R, Boehler A, Ward J, Hollander K, Sugar T, Seyfarth A 2016 A powered prosthetic ankle joint for walking and running. Biomedical Engineering Online 15(3), 141. 28105953 10.1186/s12938-016-0286-7 PMC5249039

[r21] Grimmer M, Holgate M, Ward J, Boehler A and Seyfarth A 2017 Feasibility Study of Transtibial Amputee Walking Using A Powered Prosthetic Foot, 2017 International Conference on Rehabilitation Robotics (ICORR). London: IEEE, pp. 1118–1123. Available at https://ieeexplore.ieee.org/abstract/document/8009399. 10.1109/ICORR.2017.8009399 28813971

[r22] Grimmer M, Riener R, Walsh CJ and Seyfarth A 2019a Mobility related physical and functional losses due to aging and disease-a motivation for lower limb exoskeletons. Journal of Neuroengineering and Rehabilitation 16(1), 2. 30606194 10.1186/s12984-018-0458-8 PMC6318939

[r23] Grimmer M, Schmidt K, Duarte JE, Neuner L, Koginov G and Riener R 2019b Stance and swing detection based on the angular velocity of lower limb segments during walking. Frontiers in Neurorobotics 13, 57. 31396072 10.3389/fnbot.2019.00057 PMC6667673

[r24] Holgate MA, Bohler AW and Suga TG 2008 Control Algorithms for Ankle Robots: A Reflection on the State-of-the-Art and Presentation of Two Novel Algorithms, 2008 2nd IEEE RAS & EMBS International Conference on Biomedical Robotics and Biomechatronics. Scottsdale: IEEE, pp. 97–102. Available at https://ieeexplore.ieee.org/document/4762859.

[r25] Holgate MA, Sugar TG and Bohler AW 2009 A Novel Control Algorithm for Wearable Robotics Using Phase Plane Invariants, 2009 IEEE International Conference on Robotics and Automation. Kobe: IEEE, pp. 3845–3850. Available at https://ieeexplore.ieee.org/document/5152565.

[r26] Lenzi T, Hargrove L and Sensinger J 2014a Speed-adaptation mechanism: robotic prostheses can actively regulate joint torque. IEEE Robotics & Automation Magazine 21(4), 94–107.

[r27] Lenzi T, Hargrove LJ and Sensinger JW 2014b *Minimum Jerk Swing Control Allows Variable Cadence in Powered Transfemoral Prostheses, 2014 36th Annual International Conference of the IEEE Engineering in Medicine and Biology Society*. Chicago: IEEE, pp. 2492–2495. Available at https://ieeexplore.ieee.org/document/6944128. 10.1109/EMBC.2014.6944128 25570496

[r28] Lipfert SW 2010 Kinematic and Dynamic Similarities Between Walking and Running. Hamburg: Kovač.

[r29] Nilsson J and Thorstensson A 1989 Ground reaction forces at different speeds of human walking and running. Acta Physiologica Scandinavica 136(2), 217–227. 2782094 10.1111/j.1748-1716.1989.tb08655.x

[r30] Pillai MV, Kazerooni H and Hurwich A 2011 Design of A Semi-Active Knee-Ankle Prosthesis, 2011 IEEE International Conference on Robotics and Automation. Shanghai: IEEE, pp. 5293–5300. Available at https://ieeexplore.ieee.org/document/5980178.

[r31] Prochazka A, Gillard D and Bennett DJ 1997 Positive force feedback control of muscles. Journal of Neurophysiology 77(6), 3226–3236. 9212270 10.1152/jn.1997.77.6.3226

[r32] Prochazka A, Gosgnach S, Capaday C and Geyer H 2017 Neuromuscular models for locomotion. In Bioinspired Legged Locomotion. Elsevier academic press: Cambridge, Massachusetts, United States, pp. 401–453.

[r33] Quesada RE, Caputo JM and Collins SH 2016 Increasing ankle push-off work with a powered prosthesis does not necessarily reduce metabolic rate for transtibial amputees. Journal of Biomechanics 49(14), 3452–3459. 27702444 10.1016/j.jbiomech.2016.09.015

[r34] Quinlivan B, Lee S, Malcolm P, Rossi DM, Grimmer M, Siviy C, Karavas N, Wagner D, Asbeck A, Galiana I and Walsh CJ 2017 Assistance magnitude versus metabolic cost reductions for a tethered multiarticular soft exosuit. Science Robotics 2(2), 1–10. 10.1126/scirobotics.aah4416 33157865

[r35] Rouse EJ, Hargrove LJ, Perreault EJ and Kuiken TA 2014 Estimation of human ankle impedance during the stance phase of walking. IEEE Transactions on Neural Systems and Rehabilitation Engineering 22(4), 870–878. 24760937 10.1109/TNSRE.2014.2307256 PMC5823694

[r36] Schmalz T, Blumentritt S and Jarasch R 2002 Energy expenditure and biomechanical characteristics of lower limb amputee gait: the influence of prosthetic alignment and different prosthetic components. Gait & Posture 16(3), 255–263. 12443950 10.1016/s0966-6362(02)00008-5

[r37] Schmidt K, Duarte JE, Grimmer M, Sancho-Puchades A, Wei H, Easthope CS and Riener R 2017 The myosuit: bi-articular anti-gravity exosuit that reduces hip extensor activity in sitting transfers. Frontiers in Neurorobotics 11, 57. 29163120 10.3389/fnbot.2017.00057 PMC5663860

[r38] Schuy J, Burkl A, Beckerle P and Rinderknecht S 2014 A New Device to Measure Load and Motion in Lower Limb Prosthesis—Tested on Different Prosthetic Feet, 2014 IEEE International Conference on Robotics and Biomimetics (ROBIO 2014). Bali: IEEE, pp. 187–192. Available at https://ieeexplore.ieee.org/document/7090328.

[r39] Sharbafi M, Naseri A, Seyfarth A and Grimmer M 2020 Neural control in prostheses and exoskeletons. In Powered Prostheses. Elsevier academic press: Cambridge, Massachusetts, United States, pp. 153–178.

[r40] Sharbafi MA and Seyfarth A 2015 FMCH: A New Model for Human-Like Postural Control in Walking, 2015 IEEE/RSJ International Conference on Intelligent Robots and Systems (IROS). Hamburg: IEEE, pp. 5742–5747. Available at https://ieeexplore.ieee.org/document/7354192.

[r41] Song S and Geyer H 2013 Generalization of A Muscle-Reflex Control Model to 3D Walking, 2013 35th Annual International Conference of the IEEE Engineering in Medicine and Biology Society (EMBC). Osaka: IEEE, pp. 7463–7466. Available at https://ieeexplore.ieee.org/document/6611284. 10.1109/EMBC.2013.6611284 24111471

[r42] Sup F, Varol HA, Mitchell J, Withrow T and Goldfarb M 2008 Design and Control of An Active Electrical Knee and Ankle Prosthesis, 2008 2nd IEEE RAS & EMBS International Conference on Biomedical Robotics and Biomechatronics. Scottsdale: IEEE, pp. 523–528. Available at https://ieeexplore.ieee.org/document/4762811. 10.1109/BIOROB.2008.4762811 PMC2906131 20648239

[r43] Sup F, Varol HA, Mitchell J, Withrow TJ and Goldfarb M 2009a Preliminary evaluations of a self-contained anthropomorphic transfemoral prosthesis. IEEE/ASME Transactions on Mechatronics 14(6), 667–676. 20054424 10.1109/TMECH.2009.2032688 PMC2801882

[r44] Sup F, Varol HA, Mitchell J, Withrow TJand Goldfarb M 2009b Self-Contained Powered Knee and Ankle Prosthesis: Initial Evaluation on A Transfemoral Amputee, 2009 IEEE International Conference on Rehabilitation Robotics. Kyoto: IEEE, pp. 638–644. Available at https://ieeexplore.ieee.org/document/5209625. 10.1109/ICORR.2009.5209625 PMC2836171 20228944

[r45] Thatte N and Geyer H 2016 Toward balance recovery with leg prostheses using neuromuscular model control. IEEE Transactions on Biomedical Engineering 63(5), 904–913. 26315935 10.1109/TBME.2015.2472533 PMC4854805

[r46] Ward J, Schroeder K, Vehon D, Holgate R, Boehler A and Grimmer M 2015. A Rugged Microprocessor Controlled Ankle-Foot Prosthesis for Running, 39th Annual Meeting of the American Society of Biomechanics (ASB). Columbus: BioMedical Engineering OnLine.

[r47] Windrich M, Grimmer M, Christ O, Rinderknecht S and Beckerle P 2016 Active lower limb prosthetics: a systematic review of design issues and solutions. Biomedical Engineering Online 15(3), 140. 28105948 10.1186/s12938-016-0284-9 PMC5249019

[r48] Zanotto D, Stegall P and Agrawal SK 2014 Adaptive Assist-As-Needed Controller to Improve Gait Symmetry in Robot-Assisted Gait Training, 2014 IEEE International Conference on Robotics and Automation (ICRA). Hong Kong: IEEE, pp. 724–729. Available at https://ieeexplore.ieee.org/document/6906934.

[r49] Zhao G, Sharbafi M, Vlutters M, Van Asseldonk E, Seyfarth A 2017 Template Model Inspired Leg Force Feedback Based Control Can Assist Human Walking, 2017 International Conference on Rehabilitation Robotics (ICORR). London: IEEE, pp. 473–478. Available at https://ieeexplore.ieee.org/document/8009293. 10.1109/ICORR.2017.8009293 28813865

[r50] Zhao G, Sharbafi MA, Vlutters M, van Asseldonk E, Seyfarth A 2019 Bio-inspired balance control assistance can reduce metabolic energy consumption in human walking. IEEE Transactions on Neural Systems and Rehabilitation Engineering 27(9), 1760–1769. 31403431 10.1109/TNSRE.2019.2929544

