# Supplementary material for: Neuromechanical force-based control of a powered prosthetic foot
Source: Wearable Technol. 2020 Oct 23;1:e6. doi: 10.1017/wtc.2020.6 (PMC11265316; doi:10.1017/wtc.2020.6)
Supplement: Supplementary file 1 [file wtcsup.zip › S2631717620000067sup002.pdf]

RETURN FORM: A **hand-signed** copy of this LTP must be returned by email to:
journalscopyright@cambridge.org

CAMBRIDGE UNIVERSITY PRESS

NON-EXCLUSIVE GOLD OPEN ACCESS LICENCE TO PUBLISH ("LTP")

This LTP records the terms under which the article specified below will be published in **Wearable Technologies (WTC)** (the "**Journal**"). The Journal is owned and published by the Chancellor, Masters, and Scholars of the University of Cambridge acting through its department **Cambridge University Press** of University Printing House, Shaftesbury Road, Cambridge CB2 8BS, UK (the "**Publisher**").

**THE ARTICLE**	*Please insert the full title of the article below.*
**Article Title*:**	Neuromechanical Force-Based Control of a Powered Prosthetic Foot<br>(the "**Contribution**")

This LTP can be used where a Contribution has one or more authors. The sole author (or the lead author, if applicable) must complete the box below and sign this LTP on behalf of themself (and all other authors, if any).

**LEAD AUTHOR'S DETAILS AND SIGNATURE**			
**Full Legal Name*:**	Maziar Ahmad Sharbafi<br>(the "**Lead Author**")		
**Authority to sign:**	By signing this LTP, I confirm and agree that:<br>i. All information that I have entered into this LTP is correct at the time of signature.<br>ii. **EITHER**, I am the sole author and owner of the copyright in the Contribution and I agree to the terms and conditions in this LTP.<br>iii. **OR**, the copyright in the Contribution is jointly owned by me and the Author(s) *listed below* and I agree to (and am authorized by each Author to agree to) the terms of this LTP on behalf of all Authors;<br>iv. **AND**, no other person nor entity has any copyright interest in the Contribution.		
**Signature*:**	[illegible]	**Date*:**	25.8.2020

**OTHER AUTHORS' DETAILS**				
*If the Contribution is written by **two or more** authors and the copyright in the Contribution is **jointly** owned by them – please enter the details of all other individuals who contributed to the authoring of the Contribution in this box.*<br><br>*If necessary, please add any more authors at the end of this LTP.*	**Full Legal Name***	**Email address***	**Affiliation***	**Country of residence***
	Amirreza Naseri	n.amirreza@modares.ac.ir	Tarbiat Modares University	Iran
	Martin Grimmer	grimmer@sport.tu-darmstadt.de	TU Darmstadt	Germany
	Andre Seyfarth	seyfarth@sport.tu-darmstadt.de	TU Darmstadt	Germany
	(the Lead Author and each individual listed here and at the end of this LTP is, individually and collectively, the "**Author**"			

**CAMBRIDGE EMPLOYEE**<br>*You **must** check this box and enter details, if applicable.*	☐ One or more Authors are **employed by** Cambridge University Press <u>or</u> **are related to** a Cambridge University Press employee. Please provide names of the affected Author(s) and describe the relationship(s):

**GOLD OPEN ACCESS**	*Decide how the Contribution will be accessed by readers*
**CHOICE OF LICENCE**	By entering into this LTP the Author agrees that the VoR (defined in Clause 2.1.2) will be published on a Gold Open Access basis, under the terms of the Creative Commons licence selected below and subject to payment of an APC (defined in Clause 3.5).<br>Indicate, by checking one box below, which Creative Commons licence the VoR should be published under:<br>☐ **CC BY 4.0** (Attribution)<br>☐ **CC BY NC SA 4.0** (Attribution - Non-commercial - Share Alike)<br>■ **CC BY NC ND 4.0** (Attribution - Non-commercial - No Derivatives)<br>(each a "Creative Commons Licence")<br>➢ **Please be mindful of the requirements of any funding body/ies (if applicable) when selecting a licence.**<br>➢ More details about Creative Commons Licences: https://creativecommons.org/licenses/<br>➢ If a licence is not selected, the Contribution will be published under the CC BY 4.0 licence

**SUPPLEMENTARY MATERIALS**	*Identify any additional materials to be published in association with the Contribution*
If the Author intends to submit or upload any additional materials for online publication in association with the Contribution, please indicate by	☐ **NO**, Supplementary Materials will **not** be submitted or uploaded in connection with the Contribution by the Author.<br>■ **YES**, Supplementary Materials which have been entirely created by the Author ("Original SM") will be submitted to the Publisher for publication/uploading on the same terms as the Creative Commons Licence selected.<br>☐ **YES**, Supplementary Materials which contain third-party materials ("Third-party SM") will be submitted to the Publisher for publication/uploading on the same terms as the Creative Commons Licence selected. *(Only check this box if all third-party materials can be published under the Creative Commons Licence terms.)*

PLEASE NOTE: Amended/alternative versions of this LTP will not be accepted and may delay or prevent publication

checking the applicable boxes in this section.	☐	**YES,** Supplementary Materials which contain third-party materials ("Third-party SM") will be submitted to the Publisher for publication/uploading and the Author shall include a prominent notice stating the licence terms under which those additional materials can be made available.
		(the "Supplementary Material")

1 STANDARD TERMS AND CONDITIONS

1.1 The Author hereby agrees to be bound by all terms and conditions in this LTP.

2 LICENCE

2.1 The term "**Contribution**" means the article written by the Author as identified on page one of this LTP and includes, without exception, all the following versions of the article:

2.1.1 **Accepted Manuscript ("AM")**: the version of the Contribution that has been accepted for publication. This version may include revisions resulting from peer review but may be subject to further editorial input by the Publisher.

2.1.2 **Version of Record ("VoR")**: the version of the Contribution that is formally published in the Journal. This includes any '*FirstView* article' that is formally identified as being published before the compilation of a volume or issue as long as it is citable via a permanent identifying Digital Object Identifier ("**DOI**"). This does not include any 'early release article' that has not yet been fixed by processes that are still to be applied, such as copy-editing, proof corrections, layout, and typesetting. The VoR includes any corrected or enhanced VoR.

2.2 The term "**Supplementary Material**" means any additional written or illustrative materials submitted or uploaded to the Journal by the Author for publication in connection with the Contribution. Supplementary Material does not form part of the Contribution and will be made available in association with the Contribution in online format only. Supplementary Material may be original content created by the Author ("**Original SM**") or it may be third-party material sourced and cleared in accordance with Clause 5 below by the Author ("**Third-party SM**").

2.3 In consideration of publication of the Contribution, the Author hereby grants to the Publisher:

2.3.1 a non-exclusive licence to publish, reproduce, distribute, and sell the Contribution, or any part of it, and any Supplementary Material in all forms and media and in all languages throughout the world, whether print, digital / electronic, whether now known or hereinafter invented, and to grant sublicences of all translation and subsidiary rights; and

2.3.2 a non-exclusive licence to exploit all other rights in the nature of copyright, including rental, lending and database rights and all other publishing and print on demand rights in the Contribution and any Supplementary Material.

2.4 The licences described in Clause 2.3 above shall, throughout this LTP, be referred to collectively as the "**Licence**".

2.5 The Licence shall commence upon the Publisher's formal acceptance to publish the Contribution and shall endure for the legal term of copyright in the Contribution.

2.6 The Author hereby asserts his/her/their moral right always to be identified as the author of the Contribution in accordance with the provisions of the UK Copyright, Designs and Patents Act 1988.

3 GOLD OPEN ACCESS

3.1 The Publisher will publish the VoR as part of the Journal, on a Gold Open Access basis.

3.2 "**Open Access**" means content which is distributed in digital format to the end-user without charge. Open Access *may* confer certain rights in the content to the end-user, including the ability to re-distribute it or to create derivative works from it.

3.3 For details of the Publisher's Open Access policies please follow the relevant hyperlinks at: **www.cambridge.org/openaccess**.

3.4 "**Gold Open Access**" means the Publisher will make the VoR freely accessible on the Publisher's website under the terms of the Creative Commons Licence chosen by the Author in this LTP.

3.5 "**Article Processing Charge**" or "**APC**" means the payment by the Author, or a third-party on the behalf of the Author, to the Publisher in consideration for the publication of the VoR on the Publisher's website under the terms of this LTP. The VoR will not be published until the APC is paid in full, or unless the Publisher, at its sole discretion, waives or discounts the APC or the Contribution is covered by alternative funding provided in lieu of an APC under a separate agreement between the Publisher and a relevant institution or funding body.

3.6 Where applicable, a separate invoice shall be issued to the appropriate entity for the payment of the APC and the Author is responsible for providing the Publisher with sufficient details to issue the invoice.

3.7 For the avoidance of doubt, publication of the VoR under the CC BY NC SA or CC BY NC ND Creative Commons Licences expressly does **not** permit **any** commercial reuse without permission from the Publisher. The table below summarises when and where the VoR can be posted, depending on the Creative Commons Licence chosen by the Author:

Version & Creative Commons Licence	Author(s)'s personal webpage	Author(s)'s department / institutional repository	Non - commercial subject repository	Commercial repository / social media
**VoR** (under CC BY)	From first publication	From first publication	From first publication	From first publication
**VoR** (under CC BY NC SA or CC BY NC ND)	From first publication	From first publication	From first publication	**Not permitted**

3.8 For the avoidance of doubt, any type of reuse of the VoR which is not provided for under the terms of this Clause 3 and by the chosen Creative Commons Licence shall remain subject to the Publisher's permission.

4 UNDERTAKINGS AND REPRESENTATIONS

4.1 Notwithstanding the Licence, the Author agrees and undertakes that, at the time of execution of this LTP, and thereafter until the publication of the VoR, the Contribution or any prior version of it in whole or in part, is not currently under and will not be submitted for consideration for publication in any peer-reviewed scholarly journal.

4.2 The Author hereby undertakes and represents that:

4.2.1 each named Author has full authority and power to agree to the terms in this LTP;

4.2.2 the Lead Author has full authority to execute this LTP on behalf of the Author;

4.2.3 the Contribution is original and has not been previously published in whole or in part;

4.2.4 the Contribution and any Supplementary Material contain nothing that infringes any existing copyright or licence or any other intellectual property right of any third-party;

4.2.5 the Contribution and any Supplementary Material contain nothing that breaches a duty of confidentiality or discloses any private or personal information of any person without that person's written consent;

4.2.6 all statements contained in the Contribution and any Original SM purporting to be facts are true and any formula, instruction or equivalent contained therein will not, if followed accurately, cause any injury or damage to the user;

4.2.7 the Contribution and any Supplementary Material do not contain any libellous or otherwise unlawful material, or any material which would harm the reputation of the Publisher;

4.2.8 there are no actual or apparent conflicts of interest connected to the Contribution that have not previously been declared. A conflict of interest is understood to exist if an interest (financial or otherwise) exerts or appears to exert undue influence on the analysis or conclusions in the Contribution, the choice of subject matter, or in any other way that impedes or appears to impede the Author's objectivity or independence.

4.3 In the event that the Author is in breach of any of these undertakings the Publisher shall have the right to cease making the Contribution and/or any Supplementary Material available and/or to require that the Author makes any necessary revisions to the Contribution and/or any Supplementary Material (including any factual information). Any such revisions shall be governed by this LTP.

5 THIRD-PARTY MATERIALS

5.1 The Author further confirms that for (i) any Third-party SM and (ii) any other third-party material (including but not limited to textual, illustrative, audio and video content) within the Contribution:

5.1.1 licences to re-use said content throughout the world in all languages and in all forms and media have or will be obtained from the rights-holders;

5.1.2 appropriate acknowledgement to the original source of all such materials has been made; and

5.1.3 in the case of audio/video material, appropriate release forms have been obtained from the individual(s) whose likenesses are represented in the Contribution and/or Third-party SM, as applicable.

PLEASE NOTE: Amended/alternative versions of this LTP will not be accepted and may delay or prevent publication

5.2 Copies of all licences and/or release documentation acquired in accordance with Clause 5.1 above will, on request, be forwarded to the Journal's editor prior to publication of the Contribution.

5.3 The Author must ensure that all third-party permission obtained under Clause 5.1 above allows third-party material to be included in a work licensed under the Creative Commons Licence.

6 MISCELLANEOUS

6.1 The Publisher cooperates with various copyright licensing schemes which allow material to be photocopied within agreed restraints (e.g. the Copyright Clearance Center in the U.S and the Copyright Licensing Agency in the UK). Any proceeds received by the Publisher from such licences, together with any proceeds resulting from sales of subsidiary rights in the Contribution, shall be used by the Publisher to support the continuing publication of its academic works.

6.2 The information contained in this LTP will be held for record-keeping purposes. The names of the Author may be reproduced in the Journal and provided to print and online indexing and abstracting services and bibliographic databases. The Publisher complies with applicable data protection and privacy laws in the collection, retention, storage, and use of personal data.

7 ENTIRE AGREEMENT

7.1 This LTP is made between, and contains the entire agreement between, the Publisher and the Author concerning the Contribution and supersedes all related prior agreements, arrangements and understandings (whether written or oral). No addition to or modification of any provision of this LTP shall be binding unless it is in writing and signed on behalf of the Publisher and the Author.

7.2 This LTP is governed by the law of England and Wales and is subject to the exclusive jurisdiction of the English courts.

PLEASE NOTE: Amended/alternative versions of this LTP will not be accepted and may delay or prevent publication

ADDITIONAL AUTHORS' DETAILS

If the Contribution is written by ***two or more*** *authors and the copyright in the Contribution is* ***jointly*** *owned by them – please enter the details of all other individuals who contributed to the authoring of the Contribution in this box.*

If necessary, please ***photocopy*** *this page and attach it to your LTP.*

**Full Legal Name***	**Email address***	**Affiliation***	**Country of residence***

(the Lead Author and each individual listed here and at the beginning of this LTP is, individually and collectively, the "**Author**"")

PLEASE NOTE: Amended/alternative versions of this LTP will not be accepted and may delay or prevent publication
